# Mapping Brain Motor Functions Using Transcranial Magnetic Stimulation with a Volume Conductor Model and Electrophysiological Experiments

**DOI:** 10.3390/brainsci13010116

**Published:** 2023-01-09

**Authors:** Keigo Hikita, Jose Gomez-Tames, Akimasa Hirata

**Affiliations:** 1 Department of Electrical and Mechanical Engineering, Nagoya Institute of Technology, Nagoya 466-8555, Aichi, Japan; 2 Center for Frontier Medical Engineering, Chiba University, Chiba 263-8522, Chiba, Japan

**Keywords:** brain mapping, individual head models, motor cortex, transcranial magnetic stimulation, TMS, volume conductor model

## Abstract

Transcranial magnetic stimulation (TMS) activates brain cells in a noninvasive manner and can be used for mapping brain motor functions. However, the complexity of the brain anatomy prevents the determination of the exact location of the stimulated sites, resulting in the limitation of the spatial resolution of multiple targets. The aim of this study is to map two neighboring muscles in cortical motor areas accurately and quickly. Multiple stimuli were applied to the subject using a TMS stimulator to measure the motor-evoked potentials (MEPs) in the corresponding muscles. For each stimulation condition (coil location and angle), the induced electric field (EF) in the brain was computed using a volume conductor model for an individualized head model of the subject constructed from magnetic resonance images. A post-processing method was implemented to determine a TMS hotspot using EF corresponding to multiple stimuli, considering the amplitude of the measured MEPs. The dependence of the computationally estimated hotspot distribution on two target muscles was evaluated (*n* = 11). The center of gravity of the first dorsal interosseous cortical representation was lateral to the abductor digiti minimi by a minimum of 2 mm. The localizations were consistent with the putative sites obtained from previous EF-based studies and fMRI studies. The simultaneous cortical mapping of two finger muscles was achieved with only several stimuli, which is one or two orders of magnitude smaller than that in previous studies. Our proposal would be useful in the preoperative mapping of motor or speech areas to plan brain surgery interventions.

## 1. Introduction

Transcranial magnetic stimulation (TMS) is a noninvasive technique that induces an electric current, i.e., an electric field (EF), in the brain to modulate motor and cognitive functions in various clinical applications [1,2,3,4]. The EF is considered the primary physical agents for activating brain neurons. TMS is used to identify brain structure–function relationships in fundamental neuroscience research and in clinical settings, e.g., in the preoperative mapping of motor and speech areas to plan brain surgery interventions [5,6].

It is difficult to estimate the stimulated area in the brain with a high precision using only the TMS coil position and orientation given the nonuniform current flow distribution as well as the magnetic coil design and current waveform. The EF nonuniformity is attributable to the complex anatomy of the brain and significant differences between individuals [7,8,9,10,11].

EF analysis has been conducted using volume conductor models. To replicate experimental scenarios, individual head models obtained from magnetic resonance imaging (MRI) data are used [12,13,14,15]. In such an analysis, the stimulation site was estimated in terms of individualized EF distributions. From the variation in measured motor-evoked potentials (MEPs), a small region in the hand knob of M1 was significantly related to resting or active motor thresholds [16,17]. In addition, weighting methods applied to the EF and peak MEP were used to localize the hand motor area, whose effectiveness is verified via direct electrical stimulation [18,19]. Furthermore, a sigmoidal curve representing the relationship between the induced EF and MEP in the target cortex was used to determine likely stimulation sites [20,21]. However, many stimuli were needed to estimate stimulation sites, and the spatial resolution for identifying multiple targets is limited; thus, the application of EF-based approaches in clinical setups is still challenging. 

Due to a limited resolution of localization, it has not yet been practical to apply TMS to locate the exact position of each muscle. If the locations of different muscles located close to each other can be identified simultaneously and quickly, it would be useful in preoperative diagnosis. Moreover, there is no consensus on the EF metric that should be used to estimate the preferential site of excitation (e.g., the gyral crown or sulcal wall) during brain mapping [22,23]. Thus, its effectiveness should also be revisited. 

The aim of the present study was to map two neighboring target muscles in the cortical motor area accurately and quickly based on an individualized analysis of the induced EFs. The simultaneous mapping of the cortical sites was made possible from the post-processing of the computed EFs by replicating the stimulation scenarios, i.e., the coil orientations and the positions that delivered high MEPs. The localizations were consistent with the putative sites, and a few stimuli were required, providing potential fast brain-mapping in clinical setups. In particular, the number of stimulations and MEPs needed for the localization are discussed.

## 2. Materials and Methods

### 2.1. Experiment

#### 2.1.1. Participants

Eleven healthy right-handed volunteers (9 males and 2 females; aged 22.1 ± 0.67 years) participated in this study. Participants were recruited from March 2021 to April 2022, and the experiment was conducted at the Center of Biomedical Physics and Information Technology in Nagoya Institute of Technology. According to their questionnaire responses, the participants had no contraindications to MRI scanning and TMS, did not take medication regularly, and had no history of psychiatric or neurological disease [24]. Written informed consent was obtained from each participant prior to their participation in the experiments. The study was approved by the ethical committee of Nagoya Institute of Technology (protocol code 2020-014 and date of approval 21 July 2020) and was conducted according to the Declaration of Helsinki.

#### 2.1.2. Electromyography Recording

**Figure 1:**
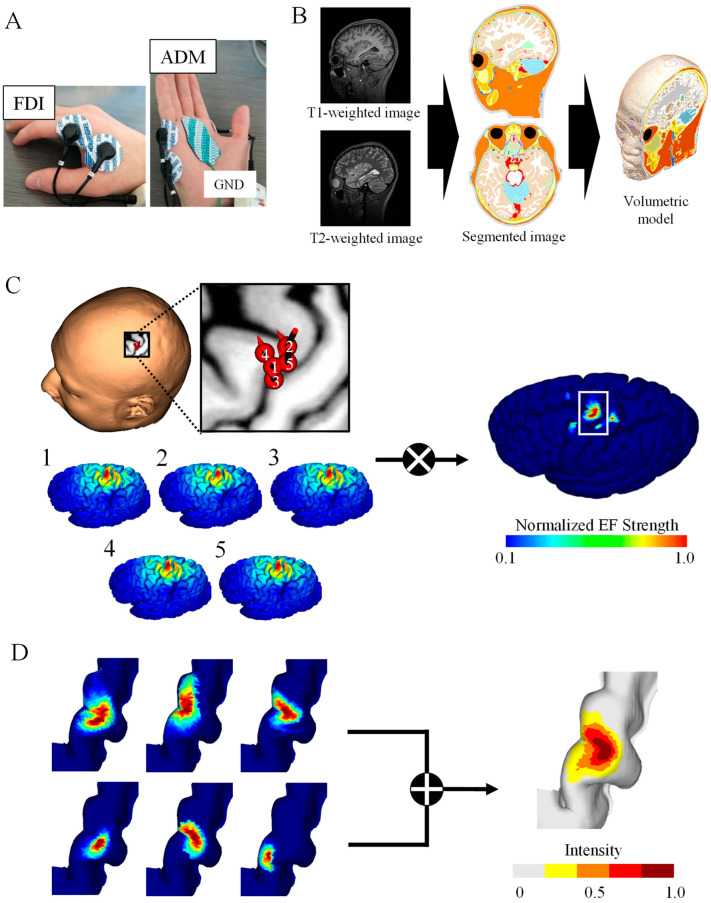
TMS brain motor mapping. (**A**) Motor-evoked potentials of two muscles were measured for a fixed simulation intensity: first dorsal interosseous (FDI) and abductor digiti minimi (ADM). (**B**) Volume conductor model from the MRI data of the participant. (**C**) Induced electric fields (EFs) on the brain cortex corresponding to the highest five MEPs were used to generate brain mapping for the FDI muscle of one participant. (**D**) Group-level hotspot identified from the linear combination of individual brain mapping for the FDI.

The participants sat on a comfortable reclining armchair during the recording of MEPs, which were recorded simultaneously from two hand muscles using a Brainsight electromyography (EMG) device (Rogue Research Inc., Montreal, QC, Canada). EMG was recorded from the first dorsal interosseous (FDI) and abductor digiti minimi (ADM), as shown in Figure 1A. The distance between the two surface electrodes of the two EMG channels was 20 mm. Data were sampled at 3 kHz and band-pass filtered between 16 and 470 Hz.

#### 2.1.3. MRI

Prior to the TMS experiment, head MRI scans were taken from each participant (T1- and T2-weighted images) using a 3 Tesla MRI. The acquisition parameters were as follows: T1 MPRAGE sequence with TR/TE/TI/FA/FOV/voxel size/slice number = 2500 ms/2.18 ms/1000 ms/8°/256 mm × 240 mm/0.8 mm × 0.8 mm × 0.8 mm/224; T2 with TR/TE/FOV/voxel size/slice number = 3200 ms/564 ms/256 mm × 240 mm/0.8 mm × 0.8 mm × 0.8 mm/224 (Figure 1B).

#### 2.1.4. TMS

Single-pulse TMS was applied to the hand motor area of the left hemisphere using a figure-8 coil (70 mm in diameter) connected to a Magstim 200^2^ magnetic stimulator (Magstim Co., Whitland, UK). The current waveform was monophasic. A TMS navigation system (Brainsight, Rogue Research Inc., Montreal, QC, Canada) was used to track and store the coil position and orientation relative to the participant’s scalp. The participants sat comfortably in a reclined chair and placed their hands on the arm rests in a relaxed posture. The participants used earplugs during the TMS operation. Because the experiment lasted approximately two hours, a 10 min break was provided between each of the three sessions described below to allow the participants to remain relaxed during the experiment.

**Figure A1:**
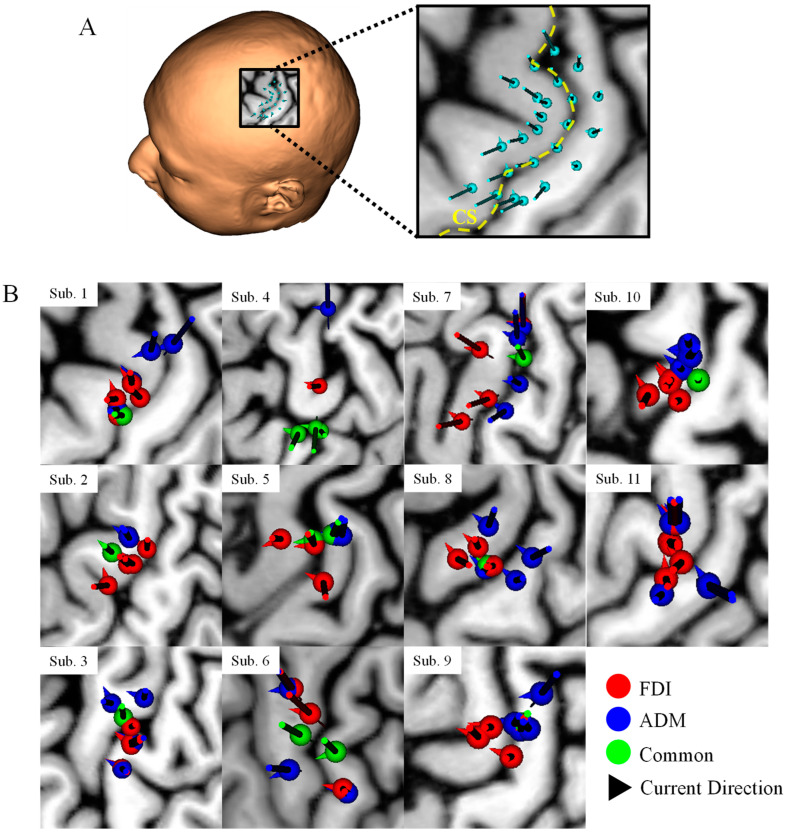
(**A**) TMS coil positions and orientations over the hand knob. The yellow dotted line indicates the central sulcus (CS). (**B**) Projection of the coil positions on the cerebral cortex that elicited the highest five MEPs for each participant. Red and blue dots represent the FDI and ADM, respectively. Green dots indicate the samples that were common to the two muscles. Arrows indicate the coil orientation. There is not clear distinction between the two target muscles based only on the coil position projection in most participants. In our study, MRI data is used as a priori information to guide the coil position and orientation (sulcus-aligned TMS coil) above the probable motor targets.

The coil was oriented to ensure that the direction of the current was perpendicular to the central sulcus (the optimal angle for the maximum EF [25]), as shown in Figure A1A. The coil placement was determined by placing eight points at 5 mm intervals along the central sulcus centered on the convexity of the hand knob, after which the coil position was shifted back and forth along the direction of the induced current at 5 mm intervals; thus, 24 stimulus points were uniquely determined for each subject. The stimulation intensity was fixed at 35%, 45%, and 55% of the maximum stimulation output of the TMS device, approximately corresponding to 83%, 106%, and 130% of the resting motor threshold (RMT), considering the different contributions of direct and indirect descending activity on MEP elicitation in the resting condition [26]. Note that, instead of RMT, the stimulation threshold is evaluated in terms of induced EF in the brain for validation, which is more relevant to the electrophysiological response (see Section 3.3).

Five MEPs were measured for each stimulation point, and 120 stimulations were performed for each intensity (4 stimuli/cm^2^). Note that a sample of 2 stimuli/cm^2^ is required for reliable mapping [19,27,28]. The order of the stimulations was randomized across participants to avoid the influence of the order of the TMS coil position on adaptation or fatigue. During the recording of the MEPs, the two muscles were in a completely relaxed state. Stimulus pulse duration is 400 μs corresponding to recruitment duration about 23 ms in MEP. The inter-trial interval varied between 5.0 s and 7.0 s to limit anticipation of the next trial. 

### 2.2. Computational Approaches

#### 2.2.1. Individualized Head Models

The head models of the participants were represented by a grid of cubical voxels with a resolution of 0.40 mm using T1- and T2-weighted images. The models were segmented into 14 tissues/body fluids (i.e., the skin, fat, muscle, outer skull, inner skull, gray matter, white matter, cerebellar gray matter, cerebellar white matter, brainstem, nuclei, ventricles, cerebrospinal fluid, and eyes). To reconstruct the surfaces of the gray and white matter, brain tissues were segmented using FreeSurfer image analysis software from T1-weighted images [29,30]. Non-brain tissues were segmented using T1- and T2-weighted images and a semi-automatic procedure which is described in more detail in [12] (Figure 1B). Note that T2 images were used to improve the quality of non-brain tissues. 

#### 2.2.2. Volume Conductor Modeling

Volume conductor modeling was applied to the head models of each participant to compute the induced EFs at different coil locations [31]. For the injection current of the TMS coils (3 kHz), magneto-quasi-static approximation was applicable because the electric displacement current was negligible compared with the conduction current and the external magnetic field was not perturbed by the induced current. The following equation was solved numerically using the finite-element method to obtain the induced scalar potential (*ϕ*):

(1) $$\nabla \cdot \sigma \nabla \varphi =-\nabla \cdot \sigma \frac{\partial A_{0}}{\partial t}$$

where A_0_ and *σ* denote the magnetic vector potential of the applied magnetic field and tissue conductivity, respectively. The magnetic vector potential A_0_ produced by the figure-8 coil was obtained using the Biot–Savart law. The coil used in the experiment was modeled via thin-wire approximation forming two wings with nine concentric loops of 9.2 cm and 5.5 cm for outer and inner loop diameters, respectively. The electric conductivity of the head tissues was assumed to be linear and isotropic, following our previous studies [19]. Note that, in this mapping, relative EF value is essential, and thus insensitive to conductivity assignment [19]. 

The TMS-induced EFs were determined from the vector potential and scalar potential using the following equation:

(2) $$\mathrm{EF}=-\nabla \varphi -\frac{\partial }{\partial t}A,$$

The computed EF and scalar electric potential corresponded to the stimulation intensity of 100% of the maximum stimulation output (%MSO), assuming a 174 A μs^−1^ peak time derivative of the coil current [17,32,33]. Then, the EFs of the experiments were obtained by scaling to the measured %MSO. The coil position and orientation were obtained from values recorded using the navigation system.

#### 2.2.3. Brain Mapping Method

Individualized induced EFs were obtained for each stimulation (*i*) with different coil orientations and positions. Cortical electric fields (*EF_i_*), sorted according to the highest peak-to-peak MEP, were multiplied to obtain the hotspot with a dominant muscle response based on the assumption that specific subareas of the hand M1 are associated with the elicitation of specific muscle movements [19]:

(3) $$EF_{focal}=\prod_{i}EF_{i},$$

The hotspot area corresponds to the cortical surface area where *EF_focal_* in Equation (3) is larger than the threshold of 0.1 × max (*EF_focal_*). Figure 1C shows the top five samples (among 120 samples) eliciting the highest MEPs in 55% MSO as well as the resulting hotspot. Detailed position and coil angle for each subject of the corresponding five samples are shown in Figure A1B. We adopted two EF metrics to identify cortical regions: the EF strength (|EF|) and normal component of the EF to the brain cortex (EF_⊥_) based on [16,17,34,35].

#### 2.2.4. Data Analysis and Stimulation Scenarios

Brain mappings of the target muscle for each individual (*n* = 11) were combined in the standard brain space (Figure 1D). For group-level analysis, the individual surface EFs were registered to the surface of the brain of the MNI ICBM 2009a standard template for each participant [12,36,37]. Group-level hotspots corresponded to the linear summation of the normalized hotspots on the standard brain template. The resulting group-level hotspot result was compared with results from previous EF-based mapping studies and functional MRI (fMRI) studies [16,38]. To make comparisons with the proposed method, the Euclidean distance of the hotspots’ center of gravity (CoG) was used.

Two metrics were considered in terms of the EF. The computed individualized |EF| or EF_⊥_ are considered to investigate the correlation with measured MEPs by fitting a sigmoid function [20]. The coefficients of determination were compared between the metrics to determine whether a more effective description of the EF related to TMS responses existed. The parameters of the sigmoid function were determined using the least-squares method:

(4) $$\mathrm{MEP}\left(EF_{max}\right)=\frac{a}{\left(1+e^{-b\left(EF_{max}-c\right)}\right)},$$

where *a* corresponds to the saturation amplitude peak-to-peak, *b* denotes the slope, and *c* denotes the location of the turning point on the abscissa. *EF_max_* represents the individual EF values at the cortical co-ordinates maximized using the localization method. The minimum EF threshold for activation was calculated as the *x*-intercept of the tangent line at the maximum point of the slope of the sigmoid function [16,39,40]. EF saturation was defined as the maximum point of the slope of the sigmoid function. Two participants were excluded from the analysis due to divergent EF saturation.

To verify the degree to which mapping accuracy can be achieved with a small amount of information, first, localization of hotspots was performed on five randomly selected samples from which a certain MEP amplitude was obtained. Thresholds for MEP amplitude were formulated in four categories (approximately 75%, 50%, 25%, and 0% of maximum MEP for suprathreshold condition). The samples that exceeded the thresholds were randomly selected to estimate the CoG. The evaluation method used was the localization error of the CoG with the localization result for the sample with the highest MEP amplitude for which there was a consensus of accuracy [19].

To evaluate errors caused by random sampling, which approximately corresponds to the uncertainty by operators, samples were selected based on random numbers. This test has been repeated 100 times.

## 3. Results

### 3.1. Estimated Hotspot Area

**Figure 2:**
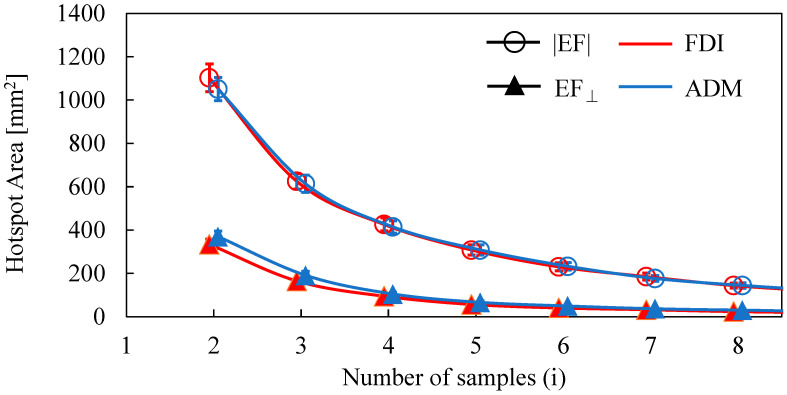
Dependence of the hotspot area on the number of samples when using the amplitude of the electric field (|EF|) and the normal component of the electric field to the wall (EF_⊥_).

The dependence of the number of samples on the estimated area of the two target muscles is shown in Figure 2. The area decreased as the number of samples was increased. The distributions of estimated areas based on the EF strength and normal component of the EF were significantly different (FDI: *p* < 0.01; ADM: *p* < 0.01). However, there was no significant difference in the estimated areas between FDI and ADM for the same EF metric (|EF|: *p* = 0.45; EF_⊥_: *p* = 0.25). Three MEP samples were needed to reach a cortical area representation of <400 mm^2^ (the estimated cortical area representation of a finger [18,19,41] for the normal component of the EF, whereas five samples were needed for EF strength.

### 3.2. Individualized and Group-Level Hotspot

**Figure 3:**
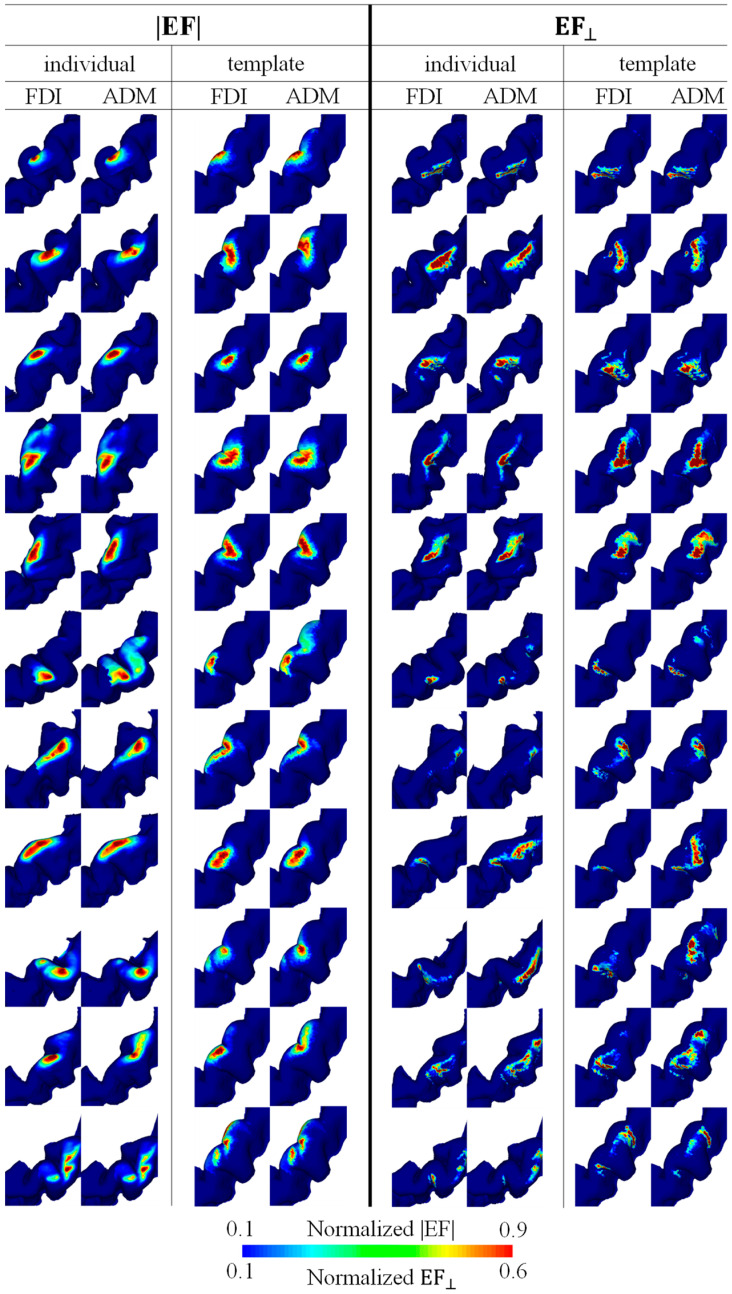
Individual brain motor mapping based on the absolute values of the electric field (|EF|) and the normal component of the electric field to the wall (EF_⊥_). Brain mappings are shown in the individual and standard brain space for each target muscle (FDI and ADM). Five and three samples were used for mapping with the |EF| and EF_⊥_, respectively.

Figure 3 shows the mapping for each individual resulting from the multiplication of EFs corresponding to the five and three highest MEPs for EF strength and the normal component of the EF, respectively. The hotspots in EF strength were observed locally in the crown of the precentral gyrus, whereas the hotspots in the normal component of the EF were at the lip of the central sulcus (see Figure 3). There was a tendency for the FDI to be lateral to the ADM, and a certain degree of overlap between the individual hotspots of the two target muscles was found in most participants.

**Figure 4:**
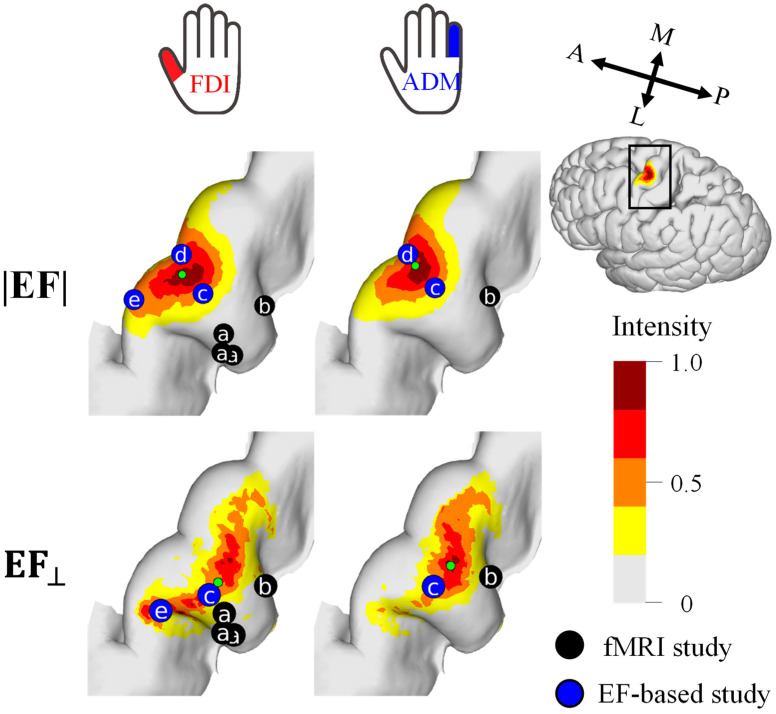
Group-level hotspot (*n* = 11) of the FDI and ADM muscles based on the electric field strength (|EF|) and normal component to the wall (EF_⊥_). Hotspots of previous fMRI studies (a [38] and b [16]) and EF-based studies (c [16], d [21], e [17]) are shown for comparison. Five and three samples were used for mapping with the |EF| and EF_⊥_, respectively.

For a quantitative analysis of the mapping results, the cortical stimulation sites were visualized at the group level (Figure 4). The cortical motor representation of the FDI was lateral to the ADM for both the EF strength and the normal component of the EF. The Euclidean distance between the CoGs of these two muscles was 1.9 mm and 3.1 mm for the EF strength and the normal component of the EF, respectively.

### 3.3. Hotspot in Randomly Selected Samples

**Figure 5:**
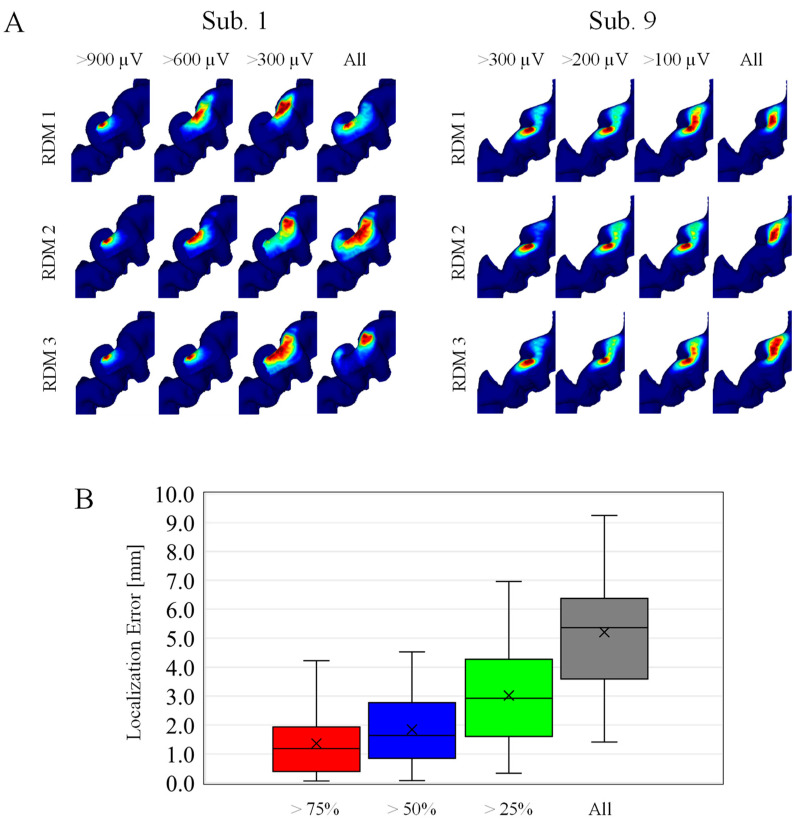
(**A**) Individualized localizations on randomly selected samples. The four columns correspond to different MEP amplitude categories. Each row corresponds to three representative cases within the 100 random (RDM) trials. (**Left**) Subjects with higher MEP response, and (**Right**) subjects with smaller MEP response at 55% MSO. (**B**) Localization errors across subjects in four different MEP amplitude categories (*n* = 100).

Samples are selected randomly based on MEP amplitude, because MEP amplitude highly depends on the individuals. For the same category, the hotspots were mapped by multiplying the corresponding EFs, and then localization accuracy is evaluated. As shown in Figure 5A, the estimated hotspot area becomes larger for estimations based on stimuli samples with a smaller MEP amplitude. As shown in Figure 5B, the localization error with an MEP response exceeding 75% and 50% of the maximum amplitude were 1.4 mm and 1.8 mm.

### 3.4. Evaluation of EF-Based Metrics

**Figure 6:**
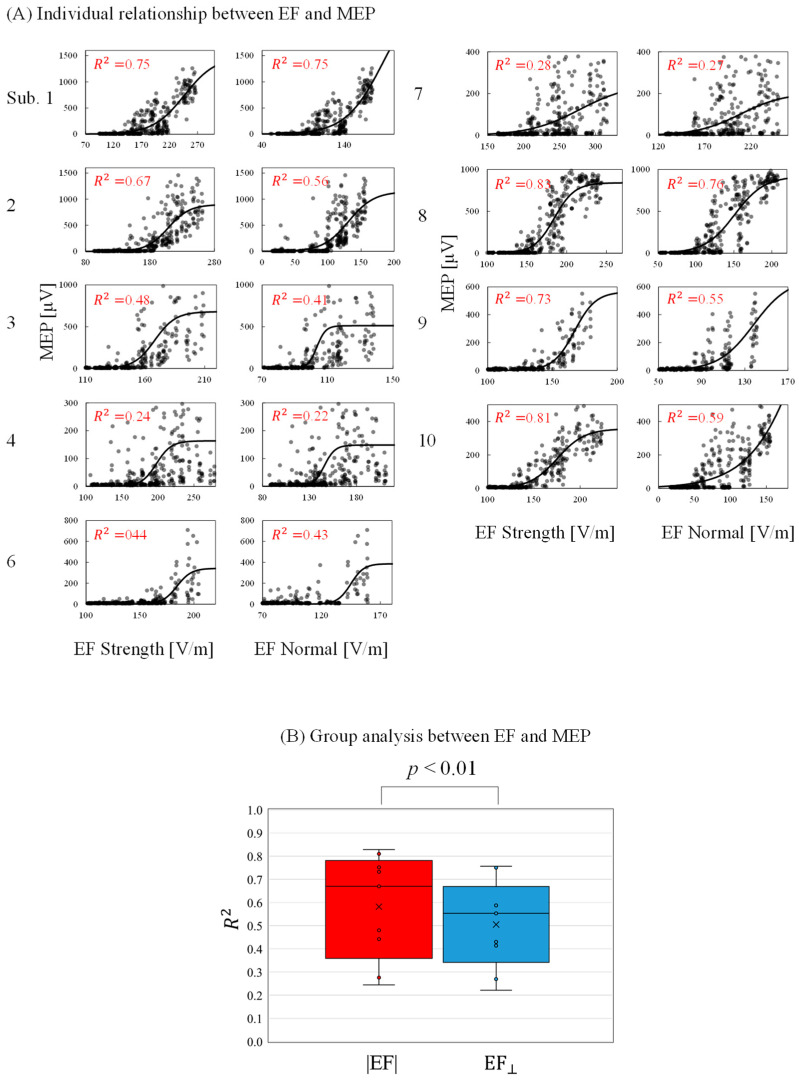
(**A**) Relationship between individual induced electric field (EF) metrics (EF strength and EF normal component) and motor-evoked potentials (MEPs) at the estimated cortical region location (coefficient of determination [*R*^2^] with *p* < 0.01 in each graph). (**B**) Grand average of the coefficient of determination (*R*^2^) between EFs and MEPs.

The relationship between the individual induced EF and measured MEPs was determined to evaluate activation characteristics and identify a preferred metric. As shown in Figure 6A, the MEP peak-to-peak values increased as the EF values increased following a sigmoidal curve. The minimum EF threshold for activation (|EF|: 174 ± 24 V/m; EF_⊥_: 127 ± 19 V/m) and the EF saturation (|EF|:235 ± 54 V/m; EF_⊥_: 190 ± 58 V/m) were determined. In addition, the RMT was determined as 42 ± 6 %MSO. The EF threshold was 5% higher than those in a previous study [17]. This difference may be attributable to the assumption of the maximum EF: this study referred to EF values 0.8 mm inside the gray matter, whereas the EF values are 2 mm in [17]. As shown in Figure 6B, the grand average of the coefficient of determination was significantly higher for the EF strength than the normal component.

## 4. Discussion

In this study, a computational method for identifying the cortical representation of muscle responses to TMS was proposed. Only the projection of the coil position corresponding to a high MEP on the cerebral cortex cannot indicate a target area with high precision. The reason for this is that simple projection does not account for the complex anatomical brain shape affecting the induced EF distribution [16,42,43], in particular when aiming to perform high-resolution mapping with multiple targets [22] (see Figure A1).

Individualized EF-based methods were used to localize stimulated areas [16,17,18,19,21]. In addition, the relationship between MEP measurements and individual EF distribution has been used with different coil positions, orientations, and intensities, which require many stimulus conditions [20]. Based on our previous study [19], we extended a method to simultaneously map the cortical sites of two finger muscles. Our proposal was based on the multiplication of the individualized induced EF strength and its normal component to the wall in the brain cortex, corresponding to stimuli with higher MEPs. One feature of our approach is that if the MEP amplitude is high enough, only three and five stimuli with high EF were required, respectively, to identify two neighboring target muscles in terms of the amplitude and normal component of EF, respectively.

Several samples were required to achieve the target areas of the FDI and ADM (Figure 2), consistent with analyses of cortical areas in a previous study [41]. In this study, the estimated area criterion is defined as <400 mm^2^. This is based on previous mapping studies, direct electric stimulation (DES) (<400 mm^2^) [18,19] and TMS (200 mm^2^) [41]. These studies confirmed that the area estimated by TMS has a more limited cortical representation than DES. This is presumably because DES can stimulate a full range of cortical representation whereas TMS is limited to areas where current can flow easily due to the complex organization of the brain. However, the nerve activation depends on the waveform of the electric current and the angle of stimulation, which should be clarified in future studies. In our measurement, about 30% of the stimuli with the highest MEPs were common to the two muscles in mapping; thus, a degree of overlap existed between the estimated hotspot areas. Nevertheless, using the CoG metric, it was possible to confirm that the FDI cortical representation was lateral to the ADM [44,45] by at least 1.9 mm. These results are in agreement with those of previous EF-based studies—(|EF|: 1.4 mm, EF_⊥_: 1.6 mm) [16] and (|EF|: 2.6 mm) [21].

According to fMRI studies, the stimulated sites activated by TMS were in the deep sulci: fMRI captures changes in blood flow during voluntary movements and the activation of proprioceptive afferent sensory feedback caused by muscle twitches. A concurrent TMS–fMRI study in which proprioceptive afferent sensory feedback of muscles were suppressed [46,47] showed that fMRI responses are close to the lip of the gyrus, which is in agreement with the results for the normal component of the EF. In the current study, there was a significant difference in the estimated distribution of the two EF metrics. Compared with EF strength, variation in the distribution of the normal component of the EF was potentially more sensitive to coil orientation, resulting in a more rapid reduction of the hotspot area according to the number of samples.

Comparison of Table 1 suggested that this method is less time-consuming than other proposed mapping approaches and will be more practical to use in clinical setups. For the post-processing of EF for three samples, it takes less than 1 min. Note that the MRI data of participants was used in the present study as a priori information to approximately guide the coil position and orientation above the probable target motor area. Although 360 samples were taken for validation, only a fractal part of them was needed. For a demonstration of high accuracy, we demonstrated samples with a larger MEP, random samples whose MEP > (50% of the max amplitude) were enough for comparable accuracy; the average MNI position was deviated less than 1.9 mm. In contrast, the distance of the representation position between the two muscles was about 1.9–3.1 mm (see Section 3.2.). Therefore, five samples with more than 50% of the maximum MEP amplitude in suprathreshold condition would be needed to estimate the target muscle with high accuracy. Assuming that we do not have any priori knowledge, i.e., if we chose five samples randomly regardless of the amplitudes, the average localization error was 5.2 mm, which is comparable to the error between the DES and the preoperative TMS (approximately 5 mm [19]). Surprisingly, even a completely random sample multiplied can estimate a position that is relatively close to the location, although inferior to samples with high MEP, suggesting its usefulness in clinical application. The point to be stressed here is that any priori knowledge was applied in this study.

The EF strength distribution was high in the crown of the gyrus, characterized by the distance from the scalp, whereas the distribution of the normal component of the EF was higher in the wall. Other studies have found a high correlation between the EF from the gyral crown and measured motor thresholds [16,20], and we found a relatively high correlation between EF strength and MEP responses (|EF|: *R*^2^ = 0.58; EF_⊥_: *R*^2^ = 0.51). A multiscale computational model revealed that stimulation sites near the crown are related to activity from interneurons, particularly for threshold conditions [35]. However, it is possible that the induced EF stimulates the superior sulci area, as shown previously via the cortical column cosine model [48], online neuroimaging during TMS [49,50], and multiscale computational analysis [23,34].

A limitation of this study is that it was conducted only with healthy young Japanese participants. Therefore, it is uncertain whether the results of this study can be directly applied to participants of different ages, races, or neurological impairments with the same accuracy. For instance, older participants have been reported to have higher motor thresholds and smaller MEP amplitudes [51]. From a racial perspective, the Japanese are known to have higher motor thresholds than Caucasians or the Chinese [52,53,54]. This is due to differences in the flow of electric currents in the brain caused by anatomical differences such as skull shape among racial groups [52]. In addition, stimulus intensity was fixed across subjects to demonstrate the simplicity of the experimental protocol, which may potentially affect the accuracy (See Figure 5B).

## 5. Conclusions

A simple and quick method for estimating the hotspot of two fingers was proposed based on the combination of individualized EFs corresponding to higher TMS-evoked MEPs according to linear sulcus-aligned TMS coil locations. The method reduces the number of required stimuli samples (an order of magnitude or more). The results of the proposed method were in good agreement with the putative locations of the target muscles and previous EF-based analysis results, which may facilitate the application of the proposed method in clinical practice.

## Figures and Tables

**Table 1:** Summary of the stimulation conditions used in previous EF-based TMS studies.

Study	Participants	TMS Type	Target	Coil Position	EF Metrics (MNI Space)
This proposal	11 healthy adults (2 females) Age: 22–24 years	Monophasic Resting Figure-8 55% MSO (suprathreshold condition)	▪FDI ▪ADM	▪Position: random ▪Angle: random (3–5 samples)	Strength (FDI: [−39, −14, 69] ADM: [−38, −15, 70]) Normal (FDI: [−42, −21, 67] ADM: [−40, −22, 69])
Bungert et al. 2017 [16]	11 healthy adults (5 females) Age: 22–24 years	Monophasic Resting Figure-8 120% rMT	▪FDI ▪ADM	▪Position: 9 × 9 grids ▪Angle: PA45° (810 samples)	Strength (FDI: [−35, −16, 61] ADM: [−34, −17, 62]) Normal (FDI: [−38, −18, 60] ADM: [−38, −18, 61])
Laakso et al. 2017 [17]	19 healthy adults (7 females) Age: 22 ± 4 years	Monophasic Active Figure-8 rMT & aMT	FDI	▪Position: 5 positions ▪Angle: PA45° (50 samples)	Strength (FDI: [−41, −7, 63]) Normal (FDI: [−43, −11, 60])
Numssen et al. 2021 [21]	14 healthy adults (7 females) Age: 21–38 years	Biphasic Resting Figure-8 120% rMT	▪FDI ▪ADM ▪APB	▪Position: random ▪Angle: random (180 samples for stableness)	Strength (FDI: [−34 −14, 67] ADM: [−33, −16, 68])

## Data Availability

The data presented in this study are contained within the article.
